# Medial gastrocnemius activation during graded axial hypergravity induced by short-arm human centrifugation

**DOI:** 10.1007/s00421-026-06332-4

**Published:** 2026-08-06

**Authors:** Chrysoula Kourtidou-Papadeli, Dimitrios Patikas, Sofia Kourtidou, Antonios Kyparos, Panagiotis Vasileiadis, Zafeiro Gkemou, Stavroula Chaloulakou, Nicholas Georgiou, Panagiotis D. Bamidis, Joan Vernikos

**Affiliations:** 1 Aeromedical Center of Thessaloniki, Thessaloniki, Greece; 2 Greek Aerospace Medical Association and Space Research, Thessaloniki, Greece; 3 Department of Physical Education and Sports Science (Serres), Aristotle University of Thessaloniki, Thessaloniki, Greece; 4 Department of Biomedical Engineering, University of Lancaster, Lancaster, USA; 5 Greek Aeropsace Medical Association and Space Research, Thessaloniki, Greece; 6 School of Medicine, Faculty of Health Sciences, National and Kapodistrian University of Athens, Athens, Greece; 7 Medical Physics and Digital Innovation Laboratory, School of Medicine, Faculty of Health Sciences, Aristotle University of Thessaloniki (AUTH), Thessaloniki, Greece; 8 Thirdage Llc, Culpeper, VA USA

**Keywords:** Artificial gravity, + Gz loading, Electromyography, Neuromuscular activation, Short-arm centrifugation, Gastrocnemius muscle

## Abstract

**Background:**

Gravitational loading is a key determinant of antigravity muscle activation, yet neuromuscular responses to graded + Gz loading in humans remain incompletely characterized. Reduced mechanical loading, whether due to microgravity or physical inactivity, leads to musculoskeletal deconditioning across both spaceflight and clinical contexts.

**Methods:**

Twenty-five adults with stable neuromuscular or neurodegenerative disorders and twenty-one age-matched healthy controls completed a short-arm human centrifugation protocol under a no-rotation baseline condition (ω = 0) and at 1.5, 1.7, and 2.0 g measured at the feet. Surface electromyography (sEMG) of the medial gastrocnemius was recorded for 60 s at each condition and analyzed as log-transformed root mean square amplitude using linear mixed-effects models.

**Results:**

Medial gastrocnemius EMG increased significantly at all hypergravity levels compared with the no-rotation baseline (main effect of condition, p ≤ 0.001). No significant main effect of group was observed, although a small group × condition interaction indicated modest differences in the magnitude of response. Within-session reliability was excellent, whereas between-day reliability was moderate. Cardiovascular parameters, assessed in a subset of participants, remained within physiological ranges across conditions.

**Conclusions:**

+ Gz loading induces reproducible increases in antigravity muscle activation in humans, in both groups of patients and healthy controls. These findings demonstrate that short-arm centrifugation provides a controllable mechanical stimulus for investigating load-dependent neuromuscular activation and human integrative responses to altered gravitational loading.

**Supplementary Information:**

The online version contains supplementary material available at https://doi.org/10.1007/s00421-026-06332-4.

## Introduction

Physical inactivity, whether due to microgravity or a sedentary lifestyle on Earth, represents a major public-health and rehabilitation challenge (Kohl et al. 2012). From a physiological perspective, altered gravitational loading provides a controlled means of modulating muscle force requirements and neural activation in humans. Although astronauts are healthy individuals, exposure to microgravity induces a state of physiological unloading that leads to rapid deconditioning of the musculoskeletal and neuromotor systems. These adaptations include reductions in muscle mass, strength, and postural control, which arise primarily from decreased gravitational loading and physical activity. Comparable physiological pathways of disuse and reduced mechanical loading are also observed in several clinical conditions associated with limited mobility or neuromuscular impairment (Narici and de Boer 2011; Hargens and Vico 2016). Therefore, while astronauts and clinical populations differ in etiology, both experience forms of unloading-induced deconditioning, making artificial gravity paradigms relevant for studying gravity-dependent neuromuscular activation and for informing rehabilitation strategies that involve controlled restoration of mechanical loading. Muscle atrophy develops when habitual loading is reduced or removed, for example, during step-reduction, immobilization, prolonged bed rest, critical illness, or aging-related sarcopenia (Bell et al. 2016; Oikawa et al. 2019; Aagaard et al. 2010). These diverse conditions converge on similar outcomes, including muscle weakness, loss of muscle mass, impaired postural control and balance, and metabolic disturbances such as insulin resistance and impaired glucose tolerance (Gopalakrishnan et al. 2010; Belavý et al. 2011; Clément 2011; Dirks et al. 2016). In neurological and neuromuscular disease, disease-specific mechanisms are superimposed on disuse, further accelerating muscle dysfunction and functional decline (Duranti and Villa 2024; Vizzi et al. 2020; Sharlo et al. 2021).

Ground-based spaceflight analogs such as long-duration head-down tilt bed rest and dry immersion reproduce key aspects of unloading-induced deconditioning and are widely used to study musculoskeletal and metabolic adaptations (Cromwell et al. 2018; Rudrappa et al. 2016; Hargens and Vico 2016; Parry and Puthucheary 2015; Clément et al. 2016). These models, together with in-flight data, show that antigravity muscles in the lower limb, including the medial gastrocnemius, undergo marked structural and functional changes during unloading and reloading (Narici and de Boer 2011; Berg et al. 1997; Fitts et al. 2010; Ohira et al. 2015; Zange et al. 1997; Trappe et al. 2009; Lambertz et al. 2001). Gravity is therefore a fundamental determinant of postural control and antigravity muscle activation (Massion 1994, 1998). Clinical assessment tools such as manual muscle testing are explicitly structured around gravitational load, and rehabilitation strategies routinely modulate effective body weight, for example through body-weight–supported treadmill training or antigravity treadmills (Avers et al. 2024; Ciesla et al. 2011; Duncan et al. 2011; Werner et al. 2002; Vincent et al. 2022).

In spaceflight and analog research, artificial gravity (AG) generated by centrifugation is actively investigated as a multisystem countermeasure to unloading (Basner et al. 2021; Convertino et al. 2001; Vernikos 1997; Clément et al. 2016; Clément and Pavy-Le Traon 2004; Rittweger et al. 2015). Short-arm centrifugation can partially mitigate musculoskeletal and sensorimotor deconditioning during head-down tilt bed rest and has been proposed as a candidate integrated countermeasure for long-duration missions (Rittweger et al. 2015; Clément et al. 2016; Bretl and Clark 2022). Experimental platforms such as the Multiple Artificial-Gravity Research System and small-animal models demonstrate that artificial 1 g or partial gravity can preserve musculoskeletal structure and function during unloading (Shiba et al. 2017; Falempin and Mounier 1998; Gao et al. 2018). Nevertheless, the optimal “dose” of AG in terms of g-level, session duration, and frequency for musculoskeletal protection and neuromuscular engagement remains unclear (Basner et al. 2021; Vernikos 1997; Bretl and Clark 2022).

Surface electromyography (sEMG) is widely used in neurorehabilitation and sports medicine as a non-invasive tool for quantifying muscle activation and providing biofeedback (Campanini et al. 2020; Haab et al. 2024; Al-Ayyad et al. 2023). Recent work has examined sEMG responses under altered loading conditions, including reduced body-weight support and hypergravity exercise, suggesting that sEMG may help quantify the gravitational stimulus required for muscle activation and guide individualized training prescriptions (Pietraszewski et al. 2024; Yang et al. 2007; Guillaud et al. 2024). In the context of short-arm human centrifugation (SAHC), therapeutic studies in multiple sclerosis indicate that AG training can modulate muscle oxygenation and may offer functional benefits, but the underlying neuromuscular activation patterns have not been fully characterized (Kourtidou-Papadeli et al. 2022; Bhambhani et al. 1998; Hiroyuki et al. 2002).

Against this background, quantifying neuromuscular responses across graded axial hypergravity conditions is relevant both for space medicine and for terrestrial rehabilitation contexts, as both involve physiological adaptations to altered gravitational loading and periods of reduced mechanical stress on the musculoskeletal system. Although astronauts are healthy individuals, exposure to microgravity produces musculoskeletal and neuromotor deconditioning that shares several physiological mechanisms with disuse and reduced loading observed in clinical populations. Therefore, artificial gravity paradigms developed for spaceflight may also provide insights relevant to rehabilitation contexts involving reduced weight bearing. The present study examined neuromuscular activation of the medial gastrocnemius during graded axial hypergravity (1.5–2.0 g) induced by SAHC in adults with neuromuscular or neurodegenerative conditions and age-matched healthy controls. We tested three hypotheses: (1) increasing + Gz exposure would produce greater medial gastrocnemius sEMG amplitude relative to the no-rotation baseline; (2) the magnitude of load-related activation would be comparable between clinical and healthy cohorts, indicating preserved relative load responsiveness; and (3) sEMG amplitude would demonstrate high within-session and acceptable between-day reliability under hypergravity conditions. By quantitatively characterizing medial gastrocnemius activation during graded SAHC, this work aims to establish SAHC as a controllable loading model for probing gravity-dependent neuromuscular recruitment in both healthy and clinical populations, without inferring functional or clinical efficacy from the present data (Basner et al. 2021; Rittweger et al. 2015; Clément et al. 2016).

## Materials and methods

### Participants

Forty-six adults participated in the study: 25 individuals with stable neuromuscular or neurodegenerative conditions and 21 age-matched healthy controls. Clinical diagnoses in the patient group included stroke, multiple sclerosis, amyotrophic lateral sclerosis, Parkinson’s disease, and muscular dystrophy (Table S1). All patients were clinically stable and under standard medical care, and none reported recent disease exacerbation.

From a pool of 35 patients, who were following a broader rehabilitation program, involving centrifugation (3 times a week for 30 min) for approximately 6 months we selected those with the most recent EMG recordings. The data captured for this study were obtained on a separate day dedicated solely to the experimental protocol. Therefore, any potential conditioning difference between the groups should be regarded as a methodological consideration rather than a primary experimental factor. All participants were able to complete the protocol with no adverse effects reported.

Healthy controls were adults with no history of neurological, cardiovascular, metabolic, or musculoskeletal disease and were not taking medications known to affect neuromuscular or cardiovascular function. Participants in both groups were not engaged in structured resistance training programs beyond standard physiotherapy.

Exclusion criteria for all participants comprised uncontrolled cardiovascular disease, severe vestibular dysfunction, epilepsy, severe migraines, cholelithiasis, kidney stones, recent surgery, acute pain or inflammation, persistent pain limiting participation, recent implantation of metal hardware or vascular stents, and any other contraindication to hypergravity exposure. Female participants provided a negative pregnancy test before enrollment. Participants taller than 2.0 m were excluded because of physical constraints related to the centrifuge arm length and positioning hardware, which limited the safe accommodation of very tall individuals on the device.

The inclusion of a heterogeneous neuromuscular cohort was intentional and aimed at examining load-related neuromuscular responsiveness across a spectrum of motor impairment rather than disease-specific mechanisms, consistent with prior work highlighting shared pathways of weakness, deconditioning, and functional decline across neurological conditions (Duranti and Villa 2024; Vizzi et al. 2020; Sharlo et al. 2021). The study was designed as a hypothesis-driven physiological investigation, not as a disease-specific clinical trial. Based on prior hypergravity and EMG studies reporting moderate within-subject effects of graded + Gz loading (Yang et al. 2007; Rittweger et al. 2015), a total sample of approximately 40–50 participants were considered sufficient to detect moderate within-subject effects in a repeated-measures design.

### Ethics

All procedures conformed to the Declaration of Helsinki and were approved by the Bioethics Committee of the School of Medicine, Aristotle University of Thessaloniki (approval no. 179/19.03.2020). Participants received verbal and written information, had the opportunity to ask questions, and provided written informed consent before participation. The intervention was registered at ClinicalTrials.gov (NCT04369976).

### Experimental design

Experiments were conducted using the GRACER short-arm human centrifuge platform developed for rehabilitation and artificial gravity research.

#### Pre-participation screening

Before the main protocol, all participants completed a 10-min familiarization trial on the short-arm human centrifuge at approximately 1 g at the feet. Individuals who experienced nausea, vertigo, or other intolerable symptoms during this trial were not enrolled. Participants were instructed to abstain from caffeine on the testing day, avoid alcohol for 12 h, refrain from eating for 2 h before the session, and avoid strenuous exercise for 24 h.

#### Short-arm human centrifuge

Centrifugation was performed using a short-arm human centrifuge (patent 1009812/13/09/2019, EP3795131) located at the Laboratory of Aerospace and Rehabilitation Applications “Joan Vernikos.” The device complies with national and international safety regulations, is certified for use in individuals with restricted mobility, and enables synchronous acquisition of physiological signals during rotation. The centrifuge has a diameter of 4 m (arm radius 2 m), accommodates two participants, and can generate up to approximately 3.5 g at the most distal part. The participants were positioned supine with their head near the axis of rotation and the feet directed outward, touching the most distal part of the arm and were secured with a harness system and lateral support to minimize body movement and head motions during centrifugation. From this position, the effective gravitational acceleration varies along the body axis as a function of distance from the axis of rotation. In the present study, + Gz levels were defined at the level of the feet, with lower acceleration experienced at more proximal anatomical levels (e.g., heart and head).

#### Experimental protocol

The centrifugation protocol was identical across both cohorts and was not modified based on clinical status. Each participant completed three experimental visits within approximately one week as part of a broader centrifuge-based intervention program. Each visit comprised 30 min of total centrifugation, delivered as five 6-min bouts separated by 4-min seated rest periods. During each visit, sEMG measurements were obtained during a standardized set of four conditions: a supine no-rotation baseline (ω = 0 rpm), followed by graded axial hypergravity at 1.5, 1.7, and 2.0 g measured at the feet. Target + Gz levels were calculated based on the centrifuge arm radius and angular velocity, with acceleration expressed relative to Earth gravity and referenced to the participant’s feet. The baseline condition corresponded to the supine no-rotation state (ω = 0), hereafter referred to as “No-rotation” in the figures.

The selected g-levels were chosen to sample a physiologically relevant range of moderate axial loading below presyncopal thresholds while respecting the mechanical and safety constraints of the centrifuge (Vernikos 1997; Clément et al. 2016; Rittweger et al. 2015; Bretl and Clark 2022). The increments between 1.5, 1.7, and 2.0 g represent small but practically achievable steps in + Gz for the given arm radius and maximum allowable rotation speed, allowing examination of neuromuscular responses across clinically and physiologically relevant hypergravity conditions without substantially prolonging exposure time or increasing the risk of motion sickness and orthostatic intolerance (Basner et al. 2021; Clément et al. 2016; Rittweger et al. 2015; Bretl and Clark 2022).

Within each bout, conditions were presented in a fixed ascending sequence from the no-rotation baseline to 1.5, 1.7, and 2.0 g for safety and operational simplicity.

For each condition, EMG acquisition began once the centrifuge reached the target + Gz and angular velocity stabilized (± 2%) and continued for 60 s. Participants were instructed to remain relaxed, to avoid voluntary contractions, and to minimize head and eye movements. Cardiovascular measurements were collected under the same gravitational conditions in a subset of participants (see below).

The medial gastrocnemius was selected as the target muscle because it is a major antigravity muscle contributing to stance and locomotion and shows pronounced structural and neuromuscular adaptations to unloading and reloading in bed-rest and spaceflight studies (Narici and de Boer 2011; Berg et al. 1997; Fitts et al. 2010; Ohira et al. 2015; Zange et al. 1997; Trappe et al. 2009).

**Fig. 1:**
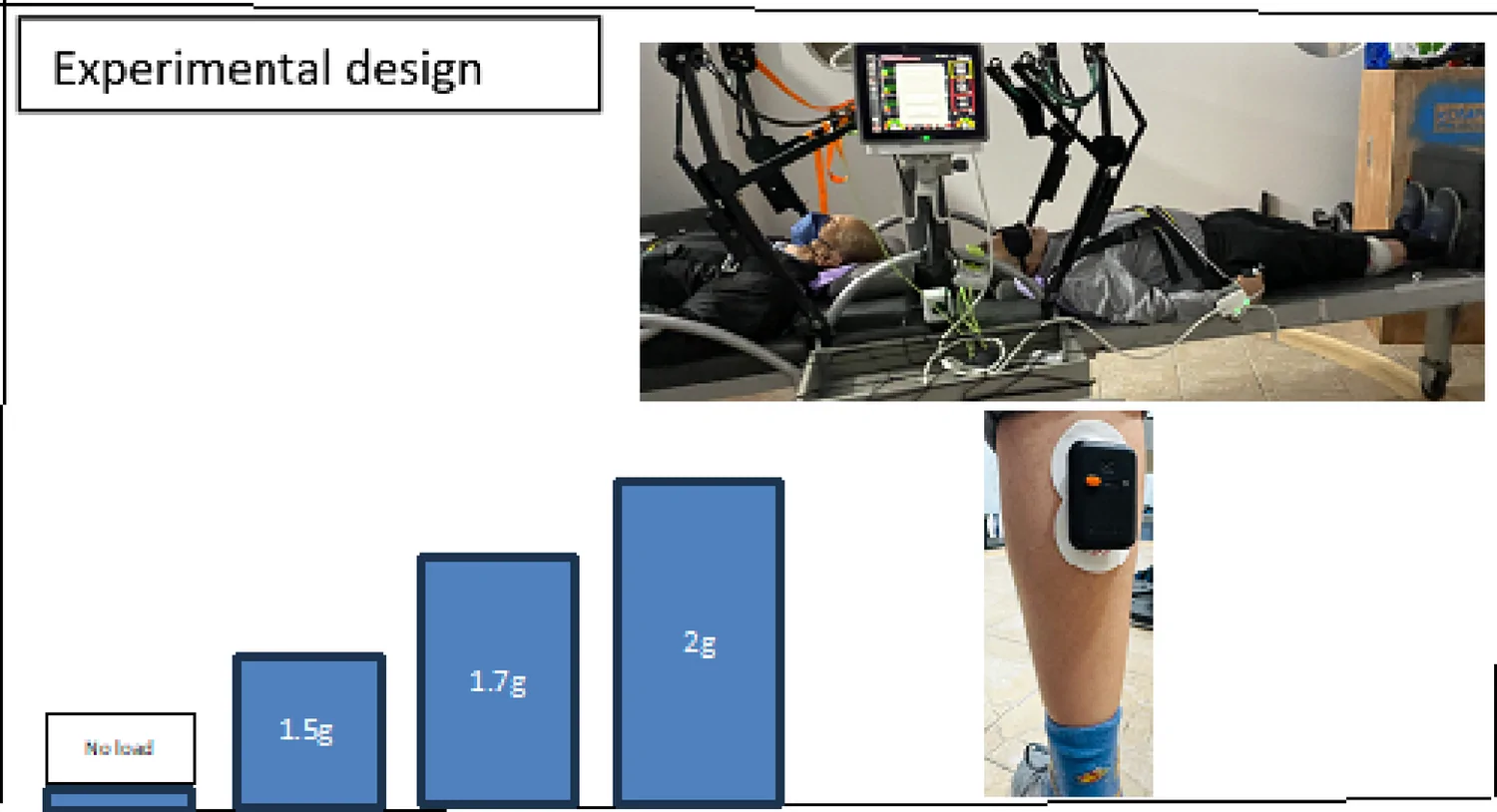
Experimental design and exposure sequence. Participants completed five 6-min bouts separated by 4-min seated rest. sEMG was recorded at no rotation (ω = 0) and at target + Gz levels of 1.5 g, 1.7 g, and 2.0 g (measured at the feet). Head and eye movements were minimized during recordings

In the figures and tables, the term “No-rotation” refers to the supine baseline condition without centrifuge rotation (ω = 0) (see Fig. 1).

#### Electromyography

Surface EMG was recorded from the medial gastrocnemius of the affected or non-dominant limb using circular pre-gelled Ag/AgCl electrodes (active diameter ≈10 mm, KFORCE EMG20012 K-MYO, KINVENT, Montpellier, France). The affected or non-dominant side was chosen to reduce potential bias related to habitual preferential use of the less affected or dominant limb. Electrode placement followed SENIAM recommendations, with an inter-electrode distance of 20 mm along the longitudinal axis of the muscle after shaving, gentle abrasion, and cleaning of the skin with 70% isopropyl alcohol (Hermens et al. 2000).

EMG signals were band-pass filtered, rectified, and expressed as root-mean-square (RMS) amplitude. EMG amplitude was analyzed in log-transformed RMS units to satisfy the assumption of normally distributed residuals. For descriptive interpretation, responses at hypergravity levels were compared with the no-rotation baseline condition obtained in the supine position (ω = 0 rpm).

Signals were amplified (× 1000), band-pass filtered between 20 and 450 Hz using a 4th-order Butterworth filter and sampled at 1 kHz. Raw data were processed offline in MATLAB (R2023b, The MathWorks Inc., Natick, MA, USA). For each 60-s recording, the first and last 10 s were discarded. The root mean square (RMS) amplitude was then calculated over the remaining 40 s using a 20-ms moving window.

For the same interval, the coefficient of variation (CV) was computed as a measure of within-trial variability according to

$$CV = {\sigma}_{RMS} / \overline{RMS } \times 100$$

where ${\sigma}_{RMS}$ and $\overline{RMS }$ denote the standard deviation and mean of the RMS EMG, respectively, between 10 and 50 s.

All EMG traces were visually inspected using custom MATLAB scripts. Segments with invalid recordings due to poor signal quality, movement artefacts, data transmission errors, or obvious phasic voluntary activation were excluded according to predefined criteria. Overall, 14.5% of trials were excluded for these reasons (Table S2). Because the study focused on within-subject changes across gravitational conditions under hypergravity, EMG amplitude was analyzed in log-transformed absolute RMS units rather than normalized to a maximal voluntary contraction, which would have been difficult to obtain safely and consistently in this heterogeneous clinical cohort (Campanini et al. 2020).

#### Cardiovascular measurements

Cardiovascular parameters were recorded in a subset of participants (15 patients, 12 controls) using a non-invasive hemodynamic monitor (ClearSight system, EV1000, Edwards Lifesciences, Irvine, CA, USA) and analyses were restricted to participants with complete datasets. The subset was determined by device availability and participant tolerance. Analyses of cardiovascular responses were restricted to individuals with complete datasets.

The ClearSight system measured cardiac output (CO, L/min), cardiac index (CI, L/min/m^2^), stroke volume (SV, mL), stroke volume index (SVI, mL/m^2^), systolic and diastolic blood pressure (SBP/DBP, mmHg), mean arterial pressure (MAP, mmHg), and heart rate (HR, beats/min). MAP was calculated as

$$MAP=\frac{SBP+2\times DBP}{3}$$

Measurements were collected every 20 s throughout each g condition. The first 30 s after reaching the target + Gz were discarded to allow hemodynamic stabilization; subsequent data points within each stage were averaged for analysis. Device calibration followed the manufacturer’s recommendations. Artefactual values and segments with signal loss were excluded. Investigators performing cardiovascular data analysis were blinded to group allocation, and participants were continuously monitored for signs of cardiovascular distress during all procedures (Basner et al. 2021).

### Statistical analysis

Repeatability of the main EMG outcome was evaluated both within sessions and across days. Within-session reliability was assessed in 21 participants with repeated trials during a single visit; between-day reliability was assessed in 28 participants who completed measurements on up to three separate days. Intraclass correlation coefficients (ICC, two-way mixed-effects model) were 0.97 within sessions and 0.53–0.55 between days, indicating excellent within-session but only moderate between-day reliability. To improve the stability of estimates and reduce the influence of day-to-day variability in electrode placement and neuromuscular state, the per-participant mean across available days was used as the primary EMG outcome for inferential analyses.

Statistical analyses were conducted in JASP (version 0.19.3, JASP Team, Amsterdam, the Netherlands) and R (version 4.4.2, R Foundation for Statistical Computing, Vienna, Austria). Log-transformed RMS EMG amplitude served as the dependent variable. Linear mixed effects models were fitted with condition (no-rotation baseline, 1.5 g, 1.7 g, and 2.0 g) and group (patients vs controls) as fixed effects, together with their interaction. Participant identity was included as a random intercept to account for repeated measurements, and additional random intercepts were included for trial and session where appropriate to capture within-subject variability. Model parameters were estimated using restricted maximum likelihood. Significance of fixed effects was assessed using F-tests with Satterthwaite or Kenward–Roger approximations for degrees of freedom, as implemented in standard mixed-model frameworks. When main effects were significant, planned contrasts compared (i) each hypergravity level with the no-rotation baseline condition and (ii) hypergravity levels with each other, within and across groups. Since EMG models were fitted on the log scale, estimated effects were back-transformed and reported as ratios or percentage changes with 95% confidence intervals (CI_95%_). Standardized effect sizes (Cohen’s d) were also calculated for key pairwise comparisons. A detailed table with all comparisons is published as supplementary material (Table S3).

For cardiovascular variables, analogous mixed-effects models were fitted with gravitational condition and group as fixed effects and participant as a random intercept. Analyses were restricted to participants with complete cardiovascular data. Fixed effects and interactions were interpreted with caution, given the smaller sample size of this subset (Basner et al. 2021). Given the repeated-measures design and large within-subject effects reported in previous hypergravity EMG studies (Yang et al. 2007; Rittweger et al. 2015), a sample of approximately 40 participants provides > 80% power to detect moderate within-subject effects.

The significance level was set at α = 0.05 for all tests. Where multiple pairwise comparisons were performed, p-values were adjusted using the Bonferroni correction. Statistical inferences are complemented by effect sizes and confidence intervals to aid interpretation.

## Results

### Participant characteristics

Forty-six participants completed the protocol (25 patients, 21 controls; mean age 51.8 ± 16.4 years). Clinical diagnoses within the patient cohort included stroke (n = 6), multiple sclerosis (n = 11), amyotrophic lateral sclerosis (n = 4), Parkinson’s disease (n = 2), and muscular dystrophy (n = 2). Baseline demographic characteristics are presented in Table 1.

**Table 1:** Basic participant characteristics

	Overall, N = 46^a^	Patients, N = 25^a^	Controls, N = 21^a^
Age (years)	51.8 ± 16.4 (41–64)	53.1 ± 14.7 (43–64)	50.3 ± 18.5 (40–62)
Height (cm)	173.2 ± 11.2 (167–180)	172.7 ± 12.1 (167–180)	173.9 ± 10.2 (167–177)
Body mass (kg)	76.6 ± 14.3 (67–85)	74.8 ± 17.0 (60–85)	78.8 ± 10.3 (75–85)
BMI^b^ (kg·m^−2^)	25.5 ± 3.7 (22–28)	24.9 ± 4.0 (22–27)	26.1 ± 3.4 (24–28)

^a^Mean ± standard deviation (Q1–Q3)

^b^Body Mass Index

### Medial gastrocnemius EMG

**Fig. 2:**
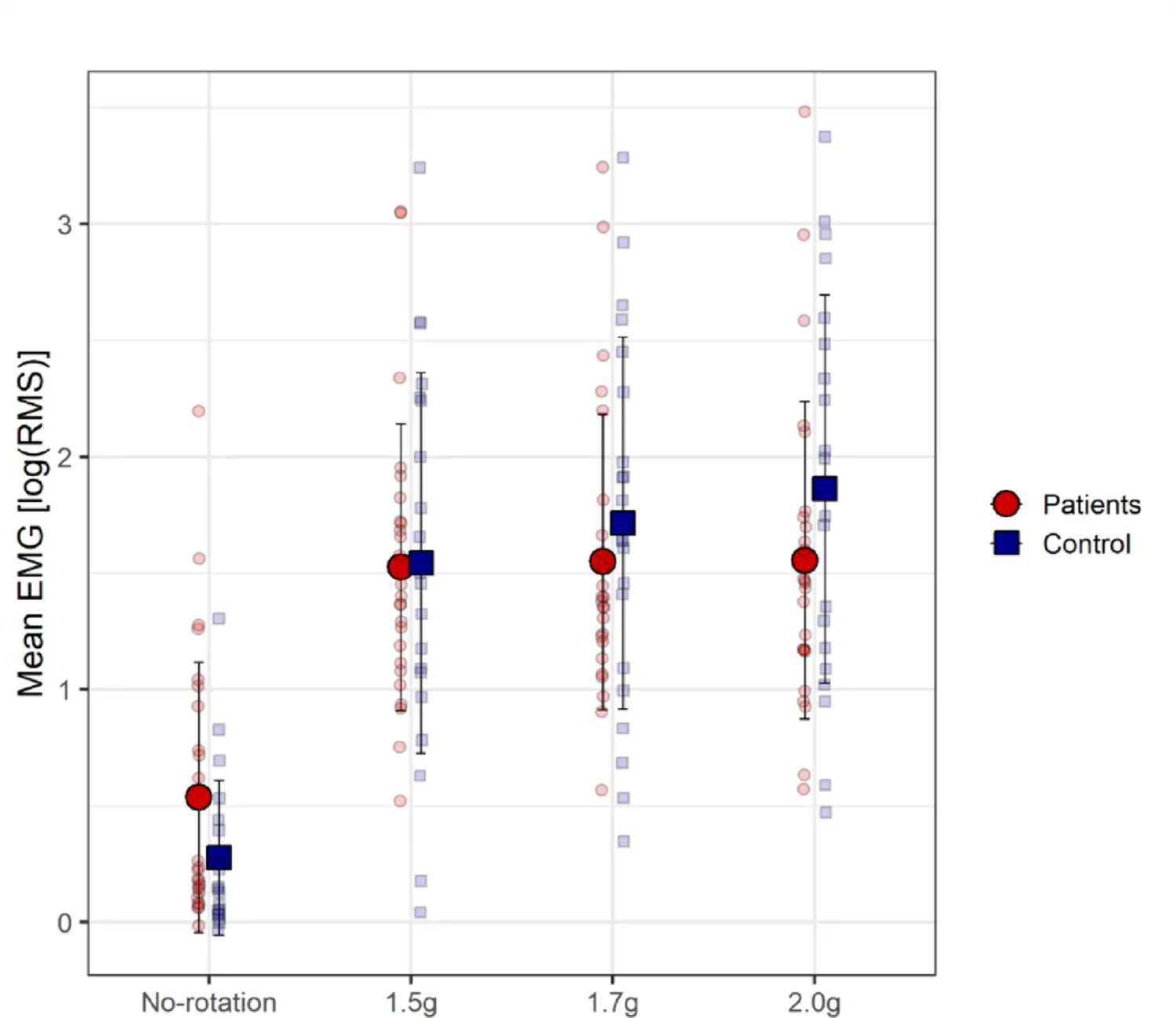
Mean EMG values during the baseline condition (“No-rotation”), corresponding to the supine no-rotation condition (ω = 0), and during exposure to 1.5 g, 1.7 g, and 2.0 g at the feet of the control group (blue) and the patients (red). Gray dots represent the individual mean values of each participant. EMG values are expressed as the log of root mean square (RMS), and vertical lines represent one standard deviation

Graded axial hypergravity significantly increased medial gastrocnemius EMG amplitude (main effect of condition: F = 81.6, p < 0.001, ηp^2^ = 0.304; Fig. 2). There was no significant main effect of group (F = 0.67, p = 0.416, ηp^2^ = 0.014), indicating similar overall EMG levels between patients and controls. However, a small but statistically significant group × condition interaction was observed (F = 2.8, p = 0.015, ηp^2^ = 0.015), suggesting modest differences in how EMG responses changed across gravitational conditions between the two groups. Using the no-rotation baseline condition as the reference, EMG amplitude was significantly elevated at 1.5 g, 1.7 g, and 2.0 g. Relative to the no-rotation baseline, medial gastrocnemius EMG increased by 155.1% in patients and by 270.9% in controls across hypergravity conditions. However, no additional pairwise comparisons between groups at individual hypergravity levels reached statistical significance. Although all hypergravity conditions differed significantly from the no-rotation baseline, differences among 1.5, 1.7, and 2.0 g were limited, indicating increased activation during centrifugation without a strictly monotonic dose-dependent relationship across the tested + Gz range. Within-session reliability of RMS amplitude was excellent (ICC = 0.95–0.97), whereas between-day reliability was moderate (ICC = 0.55).

### Cardiovascular responses

**Fig. 3:**
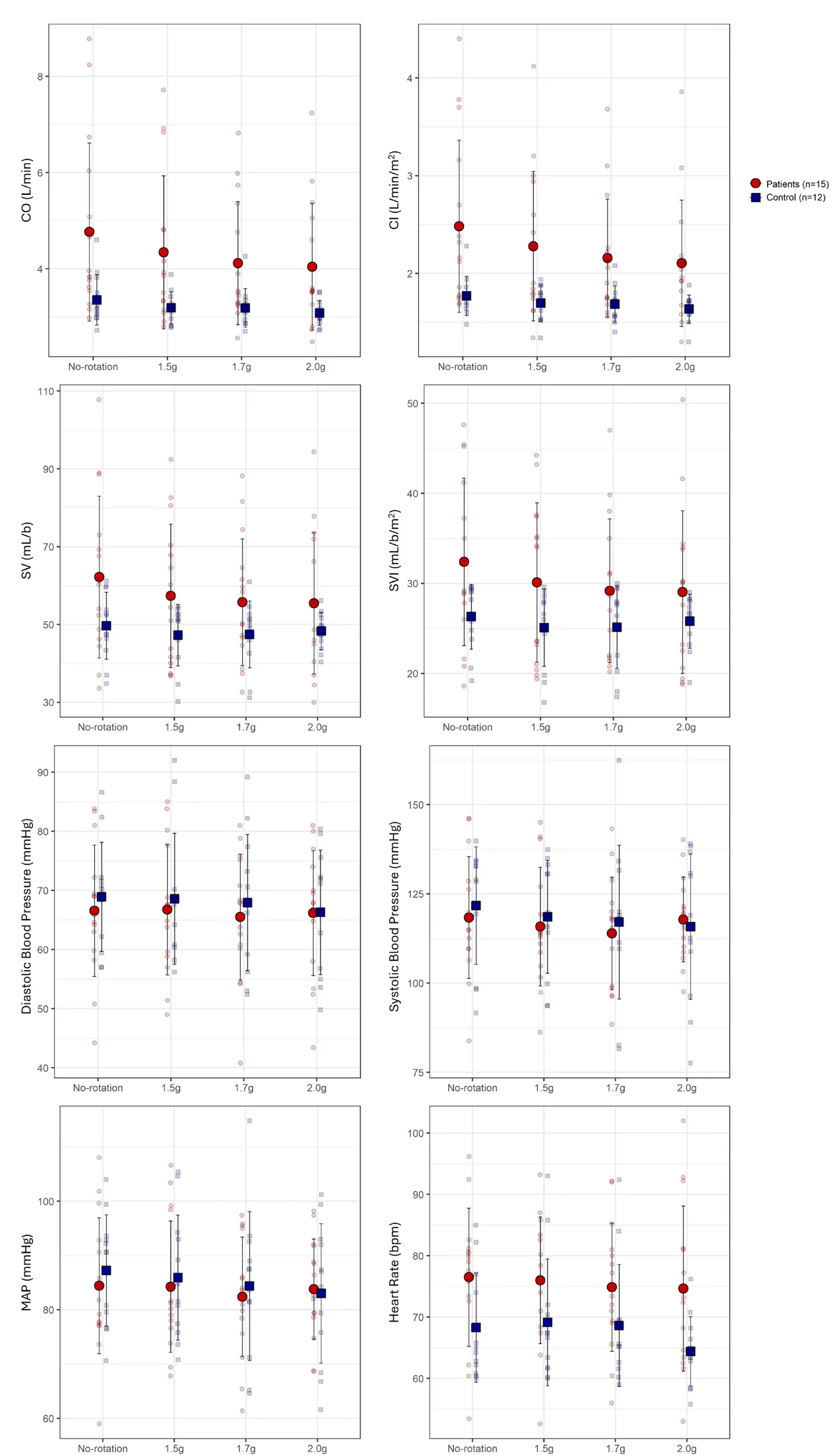
Cardiovascular responses during the baseline condition (n = 15 patients, n = 12 controls) (“No-rotation”), corresponding to the supine no-rotation condition (ω = 0), and during exposure to 1.5 g, 1.7 g, and 2.0 g at the feet. Group means ± 95% CI (colored points/bars; gray dots = individuals) for CO, CI, SV, SVI, DBP, SBP, MAP, and HR at baseline (no-rotation, ω = 0) and 1.5 g, 1.7 g, 2.0 g (g at the feet). Patients are red, controls blue

Cardiovascular measurements were available for a subset of participants (15 patients, 12 controls), and all analyses reported below are based on this subgroup. No adverse cardiovascular events occurred during centrifugation. Cardiovascular measurements were available for a subset of participants (15 patients, 12 controls), and the statistical results of the main effects and interaction are shown in Table 2. Considering the fixed effects of the linear mixed model, compared with the control group, patients had significantly higher cardiac output (1.41 ± 0.43, CI_95%_ 0.57 to 2.26 L/min, p = 0.003), cardiac index (0.71 ± 0.21, CI_95%_ 0.31 to 1.11 L/min/m^2^, p = 0.002), and heart rate (8.2 ± 3.8, CI_95%_ 0.8 to 15.7 bpm, p = 0.039). All cardiovascular variables except diastolic blood pressure were significantly affected by g-level. These effects differed between groups as revealed by the significant interaction for cardiac output (significant decrease only for patients comparing no-rotation baseline to 1.5 – 2.0 g: 0.61 ± 0.07, CI_95%_ 0.41 to 0.81 L/min, p < 0.001, d = 1.18 and comparing 1.5 g to 2.0 g: 0.32 ± 0.09, CI_95%_ 0.07 to 0.56 L/min, p = 0.003, d = 0.61), for cardiac index (significant decrease only for patients comparing no-rotation baseline to 1.5 – 2.0 g: 0.31 ± 0.03, CI_95%_ 0.20 to 0.41 L/min/m^2^, p < 0.001, d = 1.16, comparing 1.5 g to 1.7 g: 0.13 ± 0.04, CI_95%_ 0.01 to 0.25 L/min/m^2^, p = 0.046, d = 0.48 and comparing 1.5 g to 2.0 g: 0.18 ± 0.04, CI_95%_ 0.05 to 0.31 L/min/m^2^, p < 0.001, d = 0.68) and heart rate (significant decrease only for the control group comparing 1.5 g to 2.0 g: 4.7 ± 0.9, CI_95%_ 2.1 to 7.3 bpm, p < 0.001, d = 0.95 and comparing 1.7 g to 2.0 g: 4.2 ± 0.9, CI_95%_ 1.6 to 6.8 bpm, p < 0.001, d = 0.85). All other pairwise comparisons assessed did not reach the level of statistical significance (Fig. 3, Table 2).

**Table 2:** Main effects and interaction (F- p-value and partial eta squared) for 15 patients and 12 controls (subset with complete cardiovascular data) of the factor Group and G for the cardiovascular parameters

	Group	G	Group × G
CO (L/min)	**6.78, p = 0.015, ** ${{\boldsymbol{\eta}}}_{{\boldsymbol{p}}}^{2}$ ** = 0.213**	**23.90, p < 0.001, ** ${{\boldsymbol{\eta}}}_{{\boldsymbol{p}}}^{2}$ ** = 0.127**	**6.46, p < 0.001, ** ${{\boldsymbol{\eta}}}_{{\boldsymbol{p}}}^{2}$ ** = 0.038**
CI (L/min/m^2^)	**7.63, p = 0.011, ** ${{\boldsymbol{\eta}}}_{{\boldsymbol{p}}}^{2}$ ** = 0.234**	**23.79, p < 0.001, ** ${{\boldsymbol{\eta}}}_{{\boldsymbol{p}}}^{2}$ ** = 0.126**	**6.70, p < 0.001, ** ${{\boldsymbol{\eta}}}_{{\boldsymbol{p}}}^{2}$ ** = 0.039**
SV (mL/beat)	3.33, p = 0.08, ${\eta}_{p}^{2}$ = 0.118	**9.25, p < 0.001, ** ${{\boldsymbol{\eta}}}_{{\boldsymbol{p}}}^{2}$ ** = 0.053**	3.48, p = 0.016, ${\eta}_{p}^{2}$ = 0.021
SVI (mL/beat/m^2^)	3.40, p = 0.077, ${\eta}_{p}^{2}$ = 0.120	**8.93, p < 0.001, ** ${{\boldsymbol{\eta}}}_{{\boldsymbol{p}}}^{2}$ ** = 0.052**	0.33, p = 0.802, ${\eta}_{p}^{2}$ = 0.023
DBP (mmHg)	0.23, p = 0.638, ${\eta}_{p}^{2}$ = 0.009	1.93, p = 0.124, ${\eta}_{p}^{2}$ = 0.012	0.78, p = 0.508, ${\eta}_{p}^{2}$ = 0.005
SBP (mmHg)	0.13, p = 0.725, ${\eta}_{p}^{2}$ = 0.005	**3.97, p = 0.008, ** ${{\boldsymbol{\eta}}}_{{\boldsymbol{p}}}^{2}$ ** = 0.024**	1.17, p = 0.319, ${\eta}_{p}^{2}$ = 0.007
MAP (mmHg)	0.16, p = 0.691, ${\eta}_{p}^{2}$ = 0.006	**3.74, p = 0.011, ** ${{\boldsymbol{\eta}}}_{{\boldsymbol{p}}}^{2}$ ** = 0.022**	1.03, p = 0.381, ${\eta}_{p}^{2}$ = 0.006
HR (bpm)	**4.64, p = 0.041, ** ${{\boldsymbol{\eta}}}_{{\boldsymbol{p}}}^{2}$ ** = 0.157**	**8.64, p < 0.001, ** ${{\boldsymbol{\eta}}}_{{\boldsymbol{p}}}^{2}$ ** = 0.050**	**5.23, p = 0.001, ** ${{\boldsymbol{\eta}}}_{{\boldsymbol{p}}}^{2}$ ** = 0.031**

Cells in boldface indicate statistically significant effect (p < 0.05)

## Discussion

This study shows that graded axial hypergravity induced by short-arm human centrifugation increases medial gastrocnemius activation relative to the no-rotation baseline in both adults with neuromuscular or neurodegenerative conditions and healthy controls. EMG amplitude was significantly higher at 1.5, 1.7, and 2.0 g than during the no-rotation baseline, with large within-subject effects and excellent within-session reliability. Patients and controls displayed broadly similar patterns of load-related activation, although a small group × condition interaction suggested somewhat greater EMG increases at higher g levels in controls. Cardiovascular responses remained within physiological ranges without adverse events, supporting the tolerability of moderate-intensity SAHC in clinically stable adults (Basner et al. 2021; Rittweger et al. 2015; Clément et al. 2016).

### Load-dependent neuromuscular recruitment

Since gravitational loading was defined at the feet, the medial gastrocnemius likely experienced acceleration values close to the nominal + Gz levels, whereas more proximal structures, such as the heart and head were exposed to lower effective accelerations. This gradient distribution of acceleration may contribute to the observed cardiovascular responses and should be considered when comparing results with other centrifuge configurations or gravitational models.

Our findings extend previous work demonstrating that antigravity muscles are highly sensitive to changes in gravitational loading during bed rest, dry immersion, and spaceflight (Narici and de Boer 2011; Berg et al. 1997; Fitts et al. 2010; Ohira et al. 2015; Trappe et al. 2009; Lambertz et al. 2001). The pronounced increase in medial gastrocnemius EMG at all hypergravity conditions relative to the no-rotation baseline supports the concept that axial hypergravity provides a controllable mechanical stimulus capable of modulating motor unit recruitment as axial loading increases. Unlike conventional resistance exercise, the present protocol isolated gravitational loading while participants remained relaxed; the observed EMG increases, therefore, likely reflect reflex-mediated and postural neuromuscular adjustments required to stabilize the limb under increased axial loading. This supports the use of hypergravity as a model to study gravity-dependent neuromuscular control independent of task complexity or intentional contraction (Massion 1994, 1998). The present findings should therefore be interpreted primarily as evidence of load-dependent neuromuscular activation under controlled hypergravity conditions, rather than as direct evidence of functional or therapeutic efficacy, because although increased EMG amplitude reflects greater neuromuscular activation, it should not be interpreted as a direct surrogate of functional performance or clinical improvement.

The small group × condition interaction, with somewhat larger EMG increases at higher g in controls than in patients, suggests that neuromuscular impairments may modestly attenuate the magnitude of the load–activation relationship. However, the absence of a significant main effect of group and the overall similarity in responses indicate that relative load responsiveness of the medial gastrocnemius is largely preserved across this heterogeneous clinical cohort. This aligns with the notion that basic load-sensing mechanisms in muscle and peripheral reflex pathways remain operational even in the presence of neurological or neuromuscular disease, although their translation into functional performance may be compromised (Duranti and Villa 2024; Vizzi et al. 2020; Sharlo et al. 2021).

### Artificial gravity as a loading model

Artificial gravity has been widely investigated as a multisystem countermeasure for musculoskeletal, cardiovascular, and sensorimotor deconditioning during spaceflight and in ground-based analogs. Because these adaptations are largely driven by changes in gravitational loading and physical inactivity, controlled hypergravity exposure may also provide a useful experimental model for studying load-dependent neuromuscular activation in clinical populations experiencing reduced weight bearing (Basner et al. 2021; Vernikos 1997; Clément et al. 2016; Rittweger et al. 2015; Young et al. 1989). Bed rest studies using short-arm centrifugation have shown partial preservation of muscle function, balance, and bone markers, but neuromuscular activation patterns under graded hypergravity have been less well quantified (Rittweger et al. 2015; Clément et al. 2016). The present data demonstrate that supine SAHC can systematically modulate axial loading within a tolerable physiological range and generate reproducible EMG responses in a key antigravity muscle, without inducing hemodynamic instability or overt orthostatic intolerence symptoms.

From a space medicine perspective, quantifying these load-related EMG responses provides a mechanistic insight into load-dependent neuromuscular activation. Previous artificial gravity bed rest studies have demonstrated partial protection of musculoskeletal and cardiovascular function (Mulder et al. 2014; Rittweger et al. 2015). The strong main effect of condition, together with the absence of major group differences, suggests that moderate g levels in the range of 1.5–2.0 g are sufficient to produce marked changes in medial gastrocnemius activation in both healthy individuals and persons with neuromuscular disorders. However, the relatively small differences among 1.5, 1.7, and 2.0 g indicate that once a threshold of axial loading is reached, additional increases in + Gz may yield diminishing returns for this muscle under the specific supine configuration. Determining whether higher g levels, longer exposure, or different body orientations lead to more distinct neuromuscular profiles will require further work (Bretl and Clark 2022; Gao et al. 2018). Furthermore, future studies incorporating biomechanical modeling or direct estimation of segment-specific acceleration would further refine the interpretation of loading distribution along the body axis.

### Reliability and biomarker potential of sEMG

Within-session reliability of log RMS EMG was excellent, supporting the use of sEMG as a stable index of acute load-related activation during centrifugation. Between-day reliability was moderate, consistent with the known sensitivity of surface EMG to electrode placement, skin impedance, and day-to-day neuromuscular variability (Campanini et al. 2020). Averaging across available days improved stability and is appropriate for mechanistic studies focusing on the pattern of EMG responses across gravitational conditions rather than on absolute thresholds. For longitudinal or clinical applications, however, these findings highlight the importance of standardized electrode placement protocols, careful skin preparation, and possibly complementary outcome measures such as torque or functional performance.

In both rehabilitation and sports contexts, sEMG is already used for assessment and biofeedback (Haab et al. 2024; Al-Ayyad et al. 2023). In the setting of SAHC, our results suggest that sEMG could serve as a physiological biomarker of muscle engagement during AG sessions, helping to titrate g level and exposure time to reach target activation ranges while avoiding excessive load. Nevertheless, EMG amplitude remains a surrogate marker and does not directly quantify force production or functional capacity.

Any use of sEMG to guide gravitational loading should therefore be embedded within protocols that also measure strength, gait, fatigue, and patient-reported outcomes. Normalization to maximal voluntary contraction was not performed due to safety and feasibility constraints in the heterogeneous clinical cohort; therefore, analyses focused on within-subject changes across gravitational conditions, which are preserved under log-transformed RMS scaling.

### Cardiovascular stability

Cardiovascular monitoring revealed that CO and CI were higher in patients than in controls across conditions, whereas most variables, including SBP, DBP, MAP, and HR, remained within normal ranges and showed limited modulation with increasing + Gz. Modest group-specific interactions—decreases in CO and CI with higher g predominantly in patients and small HR reductions at the highest g in controls—likely reflect physiological adjustments to axial loading rather than instability per se. These findings are broadly in line with previous artificial-gravity studies reporting tolerable hemodynamic responses during intermittent centrifugation in healthy participants and clinical populations (Basner et al. 2021; Kourtidou-Papadeli et al. 2022). Taking together, they support the hemodynamic feasibility of moderate-intensity SAHC in clinically stable adults, while emphasizing the need for careful monitoring and individualized screening.

### Methodological considerations and future directions

Several methodological aspects should be considered when interpreting these results. First, the patient cohort was heterogeneous, including multiple neurological and neuromuscular diagnoses with varying severity. The study was designed to probe general load-dependent neuromuscular responsiveness rather than disease-specific mechanisms, but this heterogeneity likely increased variance and limits disease-specific conclusions. Future studies should include larger, stratified cohorts and standardized measures of functional status and medication use to assess whether diagnoses or phenotypes show distinct g-level dose–response profiles.

Second, only the medial gastrocnemius was examined, although many muscles contribute to stance and gait. Responses of proximal and other distal antigravity muscles may differ, especially given their distinct architecture and neural control (Narici and de Boer 2011; Fitts et al. 2010).

Third, the g-sequence was fixed in an ascending order, so the cumulative effects of repeated exposure and potential order effects regarding habituation, fatigue, or progressive physiological adaptation cannot be fully excluded. The fixed ascending sequence of gravitational conditions was chosen for safety and tolerability reasons, as standard practice in centrifugation protocols, particularly in clinical populations, dictates a gradual increase in + Gz. Randomized or counterbalanced g-sequences, possibly with shorter bouts or longer washout periods, should be considered in future protocols, to control for any cumulative effects of successive trials.​

Fourth, hypergravity exposure was delivered in a supine position, which simplifies cardiovascular control but may not fully replicate the neuromuscular demands of upright posture and locomotion. Combining supine SAHC with active tasks, or implementing upright centrifuge configurations where feasible, may strengthen the link between measured EMG responses and functional outcomes relevant to gait and balance (Rittweger et al. 2015; Guillaud et al. 2024). Finally, body height was restricted to less than 2.0 m for hardware and safety reasons, resulting in a relatively narrow stature range; height was therefore not modeled explicitly as a covariate.

Building on these findings, future research should incorporate pre-registered dose–response analyses, randomized or counterbalanced g-orders, multi-muscle EMG arrays, and functional endpoints such as strength, gait speed, balance, and fatigue. In clinical and spaceflight applications, integrating SAHC with active exercise and sEMG-based feedback could enable individualized AG prescriptions that are tailored to both neuromuscular engagement and cardiovascular tolerance (Vernikos 1997; Basner et al. 2021; Kourtidou-Papadeli et al. 2022). Such studies will be essential to determine whether the robust, graded EMG responses observed here translate into durable improvements in function and participation on Earth and after space missions.

### Limitations

This study was designed as a mechanistic, proof-of-concept investigation rather than a disease-specific clinical trial. The patient cohort was heterogeneous, including several neuromuscular and neurodegenerative conditions with varying severity and medication profiles, which likely increased variability and limits disease-specific inferences. Only the medial gastrocnemius was examined, so the results cannot be generalized to other antigravity muscles or to whole-body function.

Hypergravity exposure was delivered in a fixed ascending sequence and in supine position for safety, which may have introduced order effects and does not fully replicate the demands of upright posture and locomotion. Between-day reliability of sEMG was moderate, indicating sensitivity to day-to-day changes in neuromuscular state and electrode placement. However, day-averaging improved stability; it may have obscured clinically relevant variability. Finally, EMG amplitude provides an index of neural activation but does not directly measure force production, gait performance, or fatigue, and functional or patient-reported outcomes were not assessed.

Short-arm centrifugation also introduces Coriolis forces during head movements, which may influence sensorimotor responses; however, head position was kept stable during measurements to minimize these effects (Clément et al. 2016; Basner et al. 2021).

Future studies should therefore employ randomized or counterbalanced g-orders, include multi-muscle EMG and direct functional measures, and use larger, diagnosis-stratified cohorts to test pre-registered dose–response hypotheses.

## Conclusion

Short-arm human centrifugation at 1.5–2.0 g elicits robust increases in medial gastrocnemius activation relative to the no-rotation baseline in adults with neuromuscular or neurodegenerative conditions and in healthy controls, with excellent within-session reliability and broadly preserved relative load responsiveness across cohorts. Cardiovascular responses to graded axial hypergravity remained within physiological ranges, supporting the hemodynamic tolerability of moderate-intensity SAHC in clinically stable adults.

These findings support SAHC as a controllable experimental platform for probing load-dependent increase relative to baseline neuromuscular recruitment and informing artificial gravity–based loading strategies, while recognizing that functional outcomes and clinical efficacy were not evaluated in this study. Larger, randomized trials incorporating multi-muscle EMG, strength, and gait assessments, and patient-reported outcomes will be essential to determine whether SAHC-based protocols can translate into meaningful rehabilitation or post-flight reconditioning benefits (Rittweger et al. 2015; Clément et al. 2016; Kourtidou-Papadeli et al. 2022). Together, these findings suggest that surface EMG may serve as a practical physiological indicator of neuromuscular loading during artificial gravity exposure.

## Supplementary Information

Below is the link to the electronic supplementary material.

Supplementary Material 1

Supplementary Material 2

Supplementary Material 3

## Data Availability

The data supporting the findings of this study are available from the corresponding author upon reasonable request. The data are not publicly available due to ethical and privacy restrictions related to clinical participant information.
