# Supplementary material for: Medial gastrocnemius activation during graded axial hypergravity induced by short-arm human centrifugation
Source: Eur J Appl Physiol. 2026 Aug 6;126(11):6177–89. doi: 10.1007/s00421-026-06332-4 (PMC13751708; doi:10.1007/s00421-026-06332-4)
Supplement: Supplementary file 1 [file 421_2026_6332_MOESM1_ESM.docx]

**Supplementary Table S1:** Clinical characteristics of patient cohort (n = 25)

| **Diagnosis** | **n** | **Functional status*** | **Medication category**** |
| --- | --- | --- | --- |
| Multiple sclerosis | 11 | Ambulatory / Mild–moderate impairment | Immunomodulatory agents |
| Stroke | 6 | Mild–moderate motor impairment | Antiplatelet / antihypertensive |
| Amyotrophic lateral sclerosis | 4 | Moderate impairment | Riluzole / supportive |
| Parkinson’s disease | 2 | Ambulatory with symptoms | Dopaminergic therapy |
| Muscular dystrophy | 2 | Variable impairment | Supportive |
| * Functional status classified based on clinical assessment and ability to participate in protocol  ** Medication categories reported at a group level (not individual-level dosing) | | | |
