# Supplementary material for: Medial gastrocnemius activation during graded axial hypergravity induced by short-arm human centrifugation
Source: Eur J Appl Physiol. 2026 Aug 6;126(11):6177–89. doi: 10.1007/s00421-026-06332-4 (PMC13751708; doi:10.1007/s00421-026-06332-4)
Supplement: Supplementary file 2 [file 421_2026_6332_MOESM2_ESM.docx]

**Supplementary Table S2:** Excluded EMG trials (%) by group and condition.

| **Condition** | **Patients** | **Controls** | **Total** |
| --- | --- | --- | --- |
| **No-rotation** | 12.8 | 10.3 | 11.7 |
| **1.5 g** | 16.3 | 13.9 | 15.2 |
| **1.7 g** | 16.8 | 15.3 | 16.1 |
| **2.0 g** | 14.9 | 14.9 | 14.9 |
| **Overall** | **15.2** | **13.6** | **14.5** |
