# Supplementary material for: Medial gastrocnemius activation during graded axial hypergravity induced by short-arm human centrifugation
Source: Eur J Appl Physiol. 2026 Aug 6;126(11):6177–89. doi: 10.1007/s00421-026-06332-4 (PMC13751708; doi:10.1007/s00421-026-06332-4)
Supplement: Supplementary file 3 [file 421_2026_6332_MOESM3_ESM.docx]

**Supplementary Table S3:** Detailed results of pairwise differences between conditions for the patients and the control group.

| **Variable** | **Comparison** | **Group** | **lower CI_95%_** | **upper CI_95%_** | **Cohen’s d** |
| --- | --- | --- | --- | --- | --- |
| **Electromyographic activity (%)** | No-rotation vs. Rotation | Patients | 112.8 | 205.9 | 1.54 |
|  |  | Control | 155.3 | 439.0 | 2.16 |
|  | 1.5g vs. 1.7g | Patients | -17.4 | 28.7 | 0.05 |
|  |  | Control | -24.8 | 85.4 | 0.27 |
|  | 1.5g vs. 2.0g | Patients | -17.8 | 28.0 | 0.04 |
|  |  | Control | -18.1 | 102.1 | 0.42 |
|  | 1.7g vs. 2.0g | Patients | -20.3 | 24.3 | -0.01 |
|  |  | Control | -30.6 | 71.2 | 0.14 |
| **Cardiac Output (L/min)** | No-rotation vs. Rotation | Patients | -0.807 | -0.408 | -1.18 |
|  |  | Control | -0.422 | 0.021 | -0.39 |
|  | 1.5g vs. 1.7g | Patients | -0.484 | 0.012 | -0.46 |
|  |  | Control | -0.275 | 0.268 | -0.01 |
|  | 1.5g vs. 2.0g | Patients | -0.563 | -0.068 | -0.61 |
|  |  | Control | -0.380 | 0.163 | -0.21 |
|  | 1.7g vs. 2.0g | Patients | -0.332 | 0.174 | -0.15 |
|  |  | Control | -0.376 | 0.166 | -0.20 |
| **Cardiac Index (L/min/m^2^)** | No-rotation vs. Rotation | Patients | -0.409 | -0.205 | -2.70 |
|  |  | Control | -0.210 | 0.017 | -2.21 |
|  | 1.5g vs. 1.7g | Patients | -0.254 | -0.001 | -1.76 |
|  |  | Control | -0.149 | 0.129 | -1.75 |
|  | 1.5g vs. 2.0g | Patients | -0.307 | -0.054 | -1.16 |
|  |  | Control | -0.199 | 0.079 | -0.37 |
|  | 1.7g vs. 2.0g | Patients | -0.182 | 0.076 | -0.48 |
|  |  | Control | -0.189 | 0.089 | -0.04 |

(continued)

| **Variable** | **Comparison** | **Group** | **lower CI_95%_** | **upper CI_95%_** | **Cohen’s d** |
| --- | --- | --- | --- | --- | --- |
| **Stroke Volume (mL/beat)** | No-rotation vs. Rotation | Patients | -9.39 | -3.21 | -0.79 |
|  |  | Control | -5.47 | 1.40 | -0.25 |
|  | 1.5g vs. 1.7g | Patients | -5.71 | 1.97 | -0.23 |
|  |  | Control | -3.99 | 4.42 | 0.03 |
|  | 1.5g vs. 2.0g | Patients | -6.40 | 1.27 | -0.32 |
|  |  | Control | -3.12 | 5.29 | 0.14 |
|  | 1.7g vs. 2.0g | Patients | -4.61 | 3.22 | -0.09 |
|  |  | Control | -3.34 | 5.07 | 0.11 |
| **Stroke Volume Index (mL/beat/m²)** | No-rotation vs. Rotation | Patients | -4.63 | -1.55 | -0.78 |
|  |  | Control | -2.68 | 0.74 | -0.24 |
|  | 1.5g vs. 1.7g | Patients | -2.97 | 0.86 | -0.27 |
|  |  | Control | -2.03 | 2.16 | 0.02 |
|  | 1.5g vs. 2.0g | Patients | -3.29 | 0.54 | -0.35 |
|  |  | Control | -1.38 | 2.81 | 0.18 |
|  | 1.7g vs. 2.0g | Patients | -2.27 | 1.63 | -0.08 |
|  |  | Control | -1.45 | 2.75 | 0.16 |
| **Diastolic Blood Pressure (mmHg)** | No-rotation vs. Rotation | Patients | -9.70 | 14.45 | -0.08 |
|  |  | Control | -10.24 | 13.92 | -0.20 |
|  | 1.5g vs. 1.7g | Patients | -9.50 | 14.68 | -0.22 |
|  |  | Control | -11.75 | 12.43 | -0.10 |
|  | 1.5g vs. 2.0g | Patients | -3.06 | 1.99 | -0.12 |
|  |  | Control | -4.12 | 1.49 | -0.35 |
|  | 1.7g vs. 2.0g | Patients | -4.54 | 1.73 | 0.09 |
|  |  | Control | -4.08 | 2.78 | -0.25 |

(continued)

| **Variable** | **Comparison** | **Group** | **lower CI_95%_** | **upper CI_95%_** | **Cohen’s d** |
| --- | --- | --- | --- | --- | --- |
| **Systolic Blood Pressure (mmHg)** | No-rotation vs. Rotation | Patients | -7.22 | 1.57 | -0.25 |
|  |  | Control | -9.42 | 0.34 | -0.40 |
|  | 1.5g vs. 1.7g | Patients | -7.47 | 3.45 | -0.18 |
|  |  | Control | -7.46 | 4.49 | -0.13 |
|  | 1.5g vs. 2.0g | Patients | -4.27 | 6.63 | 0.10 |
|  |  | Control | -8.73 | 3.23 | -0.24 |
|  | 1.7g vs. 2.0g | Patients | -2.38 | 8.75 | 0.28 |
|  |  | Control | -7.24 | 4.71 | -0.11 |
| **Mean Arterial Pressure (mmHg)** | No-rotation vs. Rotation | Patients | -4.17 | 1.88 | -0.15 |
|  |  | Control | -6.18 | 0.54 | -0.36 |
|  | 1.5g vs. 1.7g | Patients | -5.84 | 1.68 | -0.27 |
|  |  | Control | -5.67 | 2.57 | -0.20 |
|  | 1.5g vs. 2.0g | Patients | -4.63 | 2.88 | -0.11 |
|  |  | Control | -7.03 | 1.20 | -0.37 |
|  | 1.7g vs. 2.0g | Patients | -2.63 | 5.04 | 0.15 |
|  |  | Control | -5.48 | 2.75 | -0.18 |
| **Heart Rate (bpm)** | No-rotation vs. Rotation | Patients | -3.07 | 0.75 | -0.23 |
|  |  | Control | -3.01 | 1.23 | -0.18 |
|  | 1.5g vs. 1.7g | Patients | -3.46 | 1.29 | -0.22 |
|  |  | Control | -3.10 | 2.10 | -0.10 |
|  | 1.5g vs. 2.0g | Patients | -3.20 | 1.54 | -0.17 |
|  |  | Control | -7.31 | -2.12 | -0.95 |
|  | 1.7g vs. 2.0g | Patients | -2.17 | 2.67 | 0.05 |
|  |  | Control | -6.81 | -1.62 | -0.85 |
